# Varicella Zoster Virus-induced Acute Retinal Necrosis Following Acute Meningoencephalitis in a Patient with Presumed COVID-19

**DOI:** 10.18502/jovr.v19i4.8579

**Published:** 2024-12-31

**Authors:** Kiana Hassanpour, Faezeh Khorasanizadeh, Mahmood Nabavi, Narsis Daftarian, Alireza Ramezani

**Affiliations:** ^1^Ophthalmic Research Center, Research Institute for Ophthalmology and Vision Science, Shahid Beheshti University of Medical Sciences, Tehran, Iran; ^2^Advanced Diagnostic and Interventional Radiology Research Center (ADIR), Tehran University of Medical Sciences, Tehran, Iran; ^3^Department of Radiology, Razi Hospital, Tehran University of Medical Sciences, Tehran, Iran; ^4^Department of Infectious Disease, Imam Hossein Hospital, Shahid Beheshti University of Medical Sciences, Tehran, Iran; ^5^Arthritis Research Canada, Vancouver, British Columbia, Canada; ^6^Experimental medicine, Department of Medicine, The University of British Columbia, Faculty of Medicine, Vancouver, British Columbia, Canada; ^7^Ophthalmic Epidemiology Research Center, Research Institute for Ophthalmology and Vision Science, Shahid Beheshti University of Medical Sciences, Tehran, Iran; ^9^Kiana Hassanpour: https://orcid.org/0000-0002-1788-7352; ^10^Alireza Ramezani: https://orcid.org/0000-0002-1925-1251

**Keywords:** Acute Retinal Necrosis, Case Report, Coronavirus Disease 2019, Meningoencephalitis, Varicella Zoster Virus

## Abstract

**Purpose:**

To report the coincidence of acute retinal necrosis (ARN) syndrome following acute meningoencephalitis and presumed coronavirus disease 2019 (COVID-19) in an immunocompetent patient.

**Case Report:**

A 58-year-old female presented to our emergency department with sudden unilateral visual loss following a recent hospitalization for viral meningoencephalitis. Magnetic resonance imaging (MRI), cerebrospinal fluid (CSF) analysis, polymerase chain reaction (PCR) of the aqueous humor, reverse transcription polymerase chain reaction (RT-PCR) of the nasopharyngeal swab specimen, chest computed tomography (CT), and fundus photography were performed for the patient. Ophthalmic examination revealed severe ocular inflammation and yellowish patches of necrotizing retinitis in the right eye, compatible with the diagnosis of ARN. The result of aqueous humor PCR was positive for varicella zoster virus (VZV). The patient received a single intravitreal ganciclovir injection and 10 days of intravenous ganciclovir, followed by oral acyclovir. The patient underwent COVID-19 screening tests: while the chest CT scan showed features highly suggestive of COVID-19, the RT-PCR was negative on two occasions. Two months later, best-corrected visual acuity improved to 20/70 in the right eye, the anterior chamber reaction and keratic precipitates resolved, and the vitreous haze decreased significantly.

**Conclusion:**

A case of VZV-induced ARN following acute meningoencephalitis was observed in association with presumed COVID-19. This could be an incidental finding during the COVID-19 pandemic; however, it could also suggest that COVID-19 might trigger ARN in cases with latent herpes family viruses.

##  INTRODUCTION

Acute retinal necrosis (ARN) syndrome is characterized by yellowish patches of retinitis in the retinal midperiphery, arterial retinal vasculopathy, and ocular inflammation. The most recognized causes of ARN are varicella zoster virus (VZV) followed by herpes simplex virus (HSV) type 1 and type 2.^[1]^

There is evidence supporting the association between ARN and neurological diseases, including meningitis and encephalitis.^[2]^ It is believed that both replicating and latent viruses can enter the eye through the optic nerve after brain involvement. Encephalitis secondary to VZV leading to ARN is extremely rare, and HSV 1 and 2 are the most common causative agents in post-encephalitis ARN.^[3]^

Several neurologic manifestations including headache, nausea, vomiting, and anosmia have been concomitantly reported since the first reports of the new coronavirus disease 2019 (COVID-19) in Wuhan, China. A few cases of encephalitis have also been reported; however, the full spectrum of neurologic manifestations of COVID-19 is yet to be determined.^[4,5]^ Here, we report a case of ARN secondary to possible VZV meningoencephalitis in a patient with chest CT findings consistent with COVID-19.

**Figure 1:**
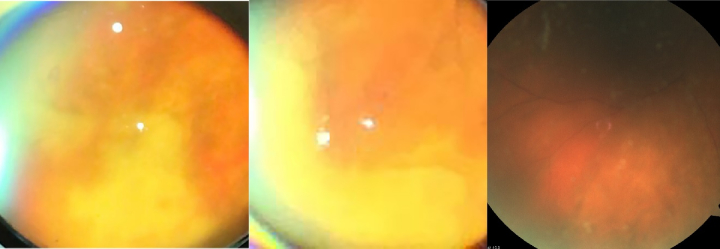
The fundus images are taken with a 20 lens and an iPhone. In the first two images from the left, one may see moderate vitreous haze and several yellowish patches with discrete borders at the far periphery of the retina, particularly in the inferior and nasal quadrants. In the third image, retinal arterial vasculitis is evident in the inferonasal branch.

**Figure 2:**
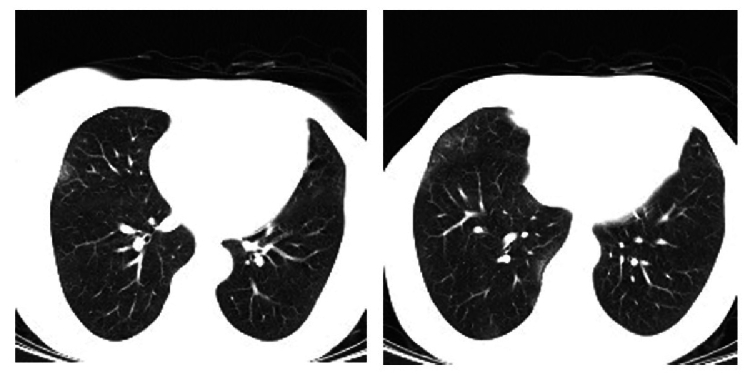
Axial chest CT shows small bilateral, multifocal peripheral and rounded ground glass opacities in the left image. The same pulmonary involvement is seen in another axial section, which is suggestive of SARS-CoV 2 infection.

##  CASE REPORT

A 58-year-old woman was referred to the ophthalmology department due to severe decline of vision in her right eye. Before this presentation, she had been hospitalized in another hospital due to a sudden-onset neurological disorder manifesting as severe headache, vomiting, and right hemiparesis. The patient had a history of chickenpox in childhood. The lumbar puncture revealed a profile suggestive of viral meningitis. The magnetic resonance imaging (MRI) showed bilateral asymmetric signal hyperintensity in the cortical and subcortical white matter of the medial temporal lobes and the left perisylvian region on fluid-attenuated inversion recovery (FLAIR) images. Although the result of polymerase chain reaction (PCR) on cerebrospinal fluid (CSF) was negative for HSV type 1 and 2, the clinical findings were highly suggestive of herpetic encephalitis. Therefore, treatment with intravenous acyclovir was started. While still in the hospital, she experienced blurred vision in her right eye and was referred for ophthalmic examination.

At presentation, best-corrected visual acuity (BCVA) was counting fingers at 4 meters in the right eye with a positive relative afferent pupillary defect. The slit-lamp biomicroscopy revealed moderate cellular reaction and flare in the anterior chamber with multiple medium-sized keratic precipitates. Signs of severe vitritis were apparent. Funduscopy revealed vitreous haze and several yellowish patches of extensive and confluent areas of necrotizing retinitis with discrete borders at the periphery of the retina, particularly in the inferior and nasal quadrants. Multiple foci of retinitis [Figure 1] as well as patches of retinal arteritis were seen posterior to the equator. The margin of the optic nerve was blurred and elevated. The results of ocular examination were unremarkable in the fellow eye.

Despite the absence of any symptoms, the patient underwent COVID-19 screening before admission in accordance with the pandemic regulations. It included chest CT and reverse transcription-PCR (RT-PCR) of the nasopharyngeal specimens. The chest CT scan revealed bilateral peripheral and rounded ground-glass opacities (GGO) that were highly suggestive of COVID-19 [Figure 2], however, the RT-PCR was negative after being repeated two times. Human immunodeficiency virus (HIV) test was negative and the CD4/CD8 ratio was normal. Multiple arms of the immune system were investigated and all tests were normal. However, the result of PCR analysis of the aqueous humor sample was positive for VZV.

Upon diagnosis with ARN, the patient received two intravitreal injections of ganciclovir (2 mg/0.1 ml, Cymevene; 500 mg; Roche, Basel, Switzerland) in addition to intravenous ganciclovir 350 mg twice daily for 10 days, followed by oral acyclovir 4 grams daily. After two days, oral prednisolone acetate 50 mg daily and aspirin 80 mg daily were prescribed for 10 days.

Two months later, BCVA reached 20/70 in the right eye. The anterior chamber reaction and keratic precipitates resolved, and the vitreous haze significantly decreased. The borders of the retinitis were sharpened and scar formation began at the edges of the improving retinitis foci. No retinal detachment or involvement of the fellow eye was observed during the two-month follow-up period.

##  DISCUSSION

ARN is mostly a clinical diagnosis with vision-threatening potential and is primarily caused by herpes viruses.^[1]^ In our case, it was caused by VZV and occurred one month after a possible viral meningoencephalitis despite the fact that the patient had received systemic acyclovir. While antiviral treatment targets actively replicating viruses, persistent latent viruses in the central nervous system (CNS) could still spread to the eye through the optic nerve at a later time, ranging from one month to several years.^[3]^

In this patient, the chest CT results were closely similar to COVID-19-induced pneumonia, but the RT-PCR test result was negative for this disease. It has been documented that the sensitivity of CT scan in diagnosing COVID-19 (98%) is higher than that of RT-PCR (71%).^[6]^ One possible explanation could be that the pneumonia was a manifestation of the patient's infection with VZV. Nevertheless, VZV pneumonia is a rare and serious complication of adult chickenpox and commonly causes numerous nodular opacities measuring 5 to 10 mm in diameter; VZV pneumonia is often accompanied by a halo sign, patchy ground-glass opacities (GGO), and diffuse coalescence of nodules.^[7]^ Therefore, in the context of the COVID-19 pandemic, VZV pneumonia diagnosis was considerably less likely in our immunocompetent, asymptomatic patient. Moreover, the peripheral features of the lesions were partially specific for COVID-19 pneumonia.^[8]^

Considering the patient's signs as a whole and the result of the aqueous humor PCR, the most probable etiology of encephalitis in our patient could be VZV. Although VZV is the most common etiologic factor for ARN, post-encephalitis ARN secondary to VZV is extremely rare. This fact is partly explained by the low incidence of encephalitis as a neurological sequela of VZV. The neurological presentations of HSV typically include encephalitis; however, VZV causes vasculopathy, hemorrhage, and necrosis in the brain rather than encephalitis.^[9,10]^

Invasion to the CNS is possible in almost all beta-coronaviruses including SARS-CoV and MERS-CoV. The entrance of the virus into the brain has been shown in experimental murine models infected with SARS-CoV and MERS-CoV. In one study, the autopsy of patients with SARS-CoV revealed the genome sequences in the brain, and the olfactory nerve was presumably the potential route of CNS involvement.^[10]^ Until the preparation of the present manuscript, a few cases of COVID-19-associated encephalitis have been reported through the pandemic.^[4,5]^

We acknowledge that our report has some limitations. First, the PCR test was negative for COVID-19, which could be attributed to the limited sensitivity of PCR testing in the early stages of the pandemic. Second, the result of CSF analysis was negative for VZV. However, considering the patient's clinical presentation, the most probable diagnosis was VZV.

In summary, we report a case of meningoencephalitis and ARN concomitant with chest CT scan findings consistent with COVID-19. This could be a coincidence of two infectious diseases in the pandemic era. Alternatively, it could also suggest the possibility that COVID-19 might trigger ARN and encephalitis in cases with latent herpes viruses. Nevertheless, this hypothesis needs to be corroborated by future investigations since COVID-19 also may cause meningoencephalitis.

##  Financial Support and Sponsorship

None.

##  Conflicts of Interest

None.
